# Total Arch With Hybrid Frozen Elephant Trunk Versus Branched Stented Anastomosis Frozen Elephant Trunk Repair

**DOI:** 10.1093/icvts/ivaf164

**Published:** 2025-07-11

**Authors:** Aakash Shah, Joshua Leibowitz, Jeffrey Lu, Douglas Tran, Julia Stallings, Shahab Toursavadkohi, Bradley Taylor, Mehrdad Ghoreishi

**Affiliations:** Department of Surgery, University of Maryland School of Medicine, Baltimore, MD, 21201, United States; Department of Surgery, University of Maryland School of Medicine, Baltimore, MD, 21201, United States; Department of Surgery, University of Maryland School of Medicine, Baltimore, MD, 21201, United States; Department of Surgery, University of Maryland School of Medicine, Baltimore, MD, 21201, United States; Department of Surgery, University of Maryland School of Medicine, Baltimore, MD, 21201, United States; Department of Surgery, University of Maryland School of Medicine, Baltimore, MD, 21201, United States; Department of Surgery, University of Maryland School of Medicine, Baltimore, MD, 21201, United States; Department of Surgery, Baptist Health, Miami, FL, 33176, United States

**Keywords:** aortic arch replacement, frozen elephant trunk, TEVAR

## Abstract

**Objectives:**

This study aims to evaluate the short-term outcomes of total arch replacement using 2 techniques: the branched stented anastomosis frozen elephant trunk repair (B-SAFER) under moderate hypothermia (25-28 °C), and a simplified total arch and hybrid arch frozen elephant trunk (HA-FET) reconstruction using the Thoraflex stent graft under mild hypothermia (>32 °C).

**Methods:**

Sixty-one patients underwent total arch replacement between June 2020 and March 2024. 25 received HA-FET, and 36 received B-SAFER. Central cannulation and cerebral debranching of the innominate and left common carotid arteries were performed before circulatory arrest in both groups. Axillary cannulation led to debranching after circulatory arrest. In the HA-FET group, snares were placed circumferentially in zone 1 and zone 2 prior to circulatory arrest and deployment of FET graft; in B-SAFER, antegrade thoracic stent graft was deployed in zone 2 with left subclavian fenestration and stenting.

**Results:**

Mean age 57.4 ± 13.1 years, with 74% male. Acute type A was the pathology in 60% of HA-FET and 58% of B-SAFER patients. HA-FET had significantly shorter circulatory arrest times (9 vs 40 minutes, *P* < .001) but similar cardiopulmonary bypass and cross-clamp times. The rate of concomitant major cardiac procedure was higher in HA-FET group (13/25, 52% vs 10/36, 27%, *P* = .066). Neurologic dysfunction (4% vs 5.4%, *P* = 1) and in-hospital mortality (4% vs 8.1%, *P* = .64) were similar. No paraplegia occurred, and renal failure requiring dialysis occurred in 12% of HA-FET and 8.1% of B-SAFER patients (*P* = .68).

**Conclusions:**

Both mild hypothermic total arch with hybrid FET repair and hypothermic total arch replacement utilizing B-SAFER technique provide safe and favourable short-term outcomes. Further studies with larger cohorts and long-term follow-up are required.

## Graphical abstract

**Figure:**
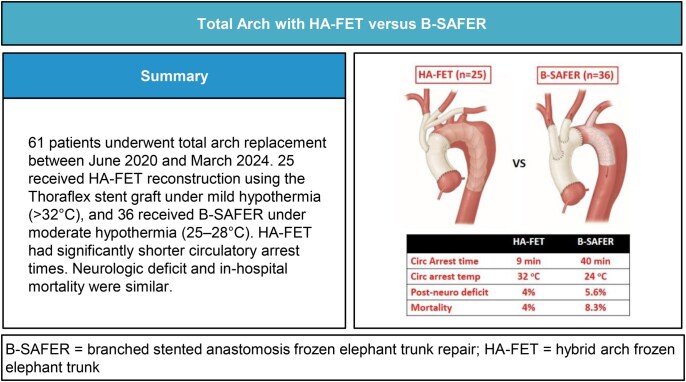

## INTRODUCTION

Total arch replacement has evolved significantly over the past decades, becoming a critical procedure in the management of complex aortic pathologies. The first successful aortic arch replacement was performed in 1957 by Dr. Michael DeBakey and his team; however, these early attempts at aortic arch repair were met with significant challenges, including high postoperative mortality and stroke rates.^1^ By the 1970s and 1980s, techniques such as deep hypothermic circulatory arrest (HCA) were developed, which involved cooling the body to a target temperature to slow metabolic activity and protect vital organs during periods of interrupted blood flow.^1^

Hypothermic circulatory arrest quickly became a cornerstone of total arch replacement, significantly reducing the risks of neurological and organ complications during surgery. However, despite its success, the risks associated with deep hypothermia, such as coagulopathy and longer recovery times, prompted a search for alternative approaches. This led to recent trends focusing on minimizing these risks by shifting towards moderate hypothermia and even normothermic techniques, aiming to reduce procedural complexity and improve patient outcomes.

The evolution of aortic arch replacement saw a significant milestone with the development of the frozen elephant trunk (FET) technique, which allows for the integration of endovascular and open surgical approaches.^2^ Initially, aortic arch surgery involved the use of separate procedures to address both the arch and the descending aorta, often requiring staged operations that increased the risk of complications and prolonged recovery times. The FET technique revolutionized this approach by combining open surgical repair of the aortic arch with endovascular stenting of the descending aorta in a single procedure.^2^

The FET technique offers several advantages, including the ability to secure the distal anastomosis in a healthier section of the aorta, reducing the risk of anastomotic complications and providing a stable landing zone for future endovascular interventions. This was further refined with the introduction of the branched stented anastomosis frozen elephant trunk (B-SAFER) technique, which involves the proximalization of the distal anastomosis from zone 3 to zone 2 of the aortic arch.^3^ By bringing the anastomosis closer to the heart, the B-SAFER technique simplifies the procedure and may offer improved outcomes, particularly in patients with challenging aortic anatomy or those at high risk for traditional open surgery.

More recently, the advent of the Thoraflex hybrid arch frozen elephant trunk (HA-FET) device (Terumo Aortic) has allowed for a streamlined approach to total arch replacement with shorter circulatory arrest time, providing the benefits of FET without the need for deep hypothermia.^4^^,^^5^ Our group adopted and modified this method to permit a more simplified delivery of an HA-FET under mild hypothermic circulatory arrest.

Having utilized both the B-SAFER and the HA-FET arch replacement techniques, we sought to compare their outcomes in terms of mortality, stroke, and other critical factors. We hypothesized that the outcomes of both methods would be equivalent, potentially providing a safer and more efficient alternative to traditional hypothermic approaches.

## METHODS

### Patients

We performed a retrospective cohort study of all subjects ≥18 years of age who underwent aortic arch replacement at University of Maryland Medical Center between June 2020 and March 2024. Patients who underwent an aortic arch replacement with a method other than B-SAFER or HA-FET were excluded from this analysis. This study was approved by the Institutional Review Board at the University of Maryland, Baltimore, who granted a waiver of written informed consent (Approved by the University of Maryland on 01/30/2020; HP-00087855).

### Surgical technique

**Figure 1.**
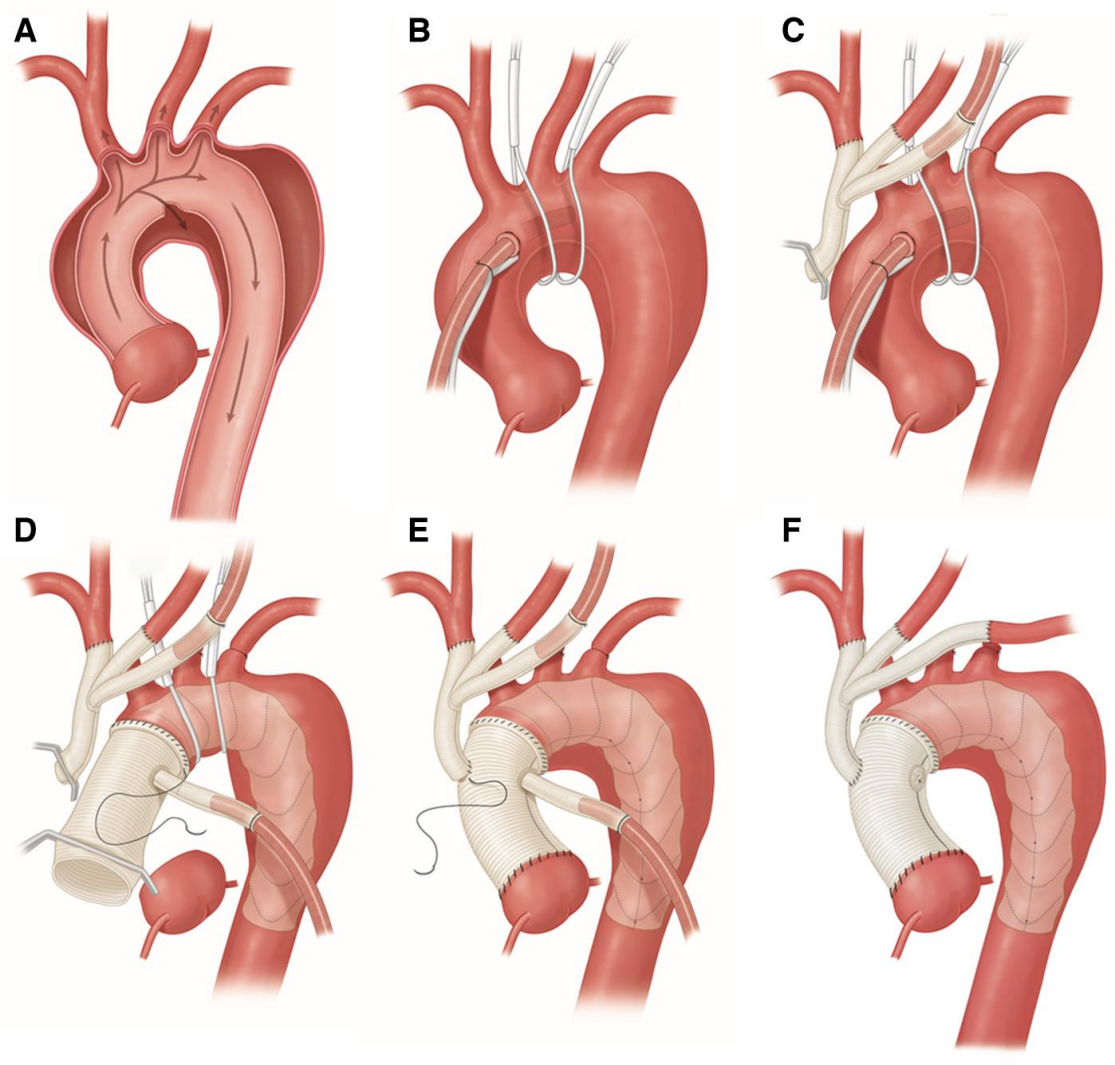
Preferred Surgical Sequence for Hybrid Arch Frozen Elephant Trunk Approach. (A) Type A aortic dissection with primary entry tear in the aortic arch. (B) Central cannulation and placement of snares circumferentially around the aortic arch in zone 1 and zone 2. (C) Cerebral debranching of the innominate and left common carotid arteries with the use of a trifurcated graft. Cerebral flow is provided by a second arterial cannula in the third limb of the branched graft and the left subclavian artery is ligated. (D) The Thoraflex is deployed in zone 0, the snares are tightened down on the stent graft portion, an arterial cannula is inserted in the perfusion limb of the Thoraflex to provide systemic flow while the distal anastomosis is completed. Bilateral antegrade cerebral perfusion is provided via the second arterial cannula in the third limb of the trifucated graft. (E) After completion of the proximal anastomosis, the common trunk of the trifurcated graft is anastomosis to the main ascending graft. (F) The arterial cannula is removed from the third limb and the left subclavian artery is then anastomosed. This depicts the completed surgery

**Figure 2.**
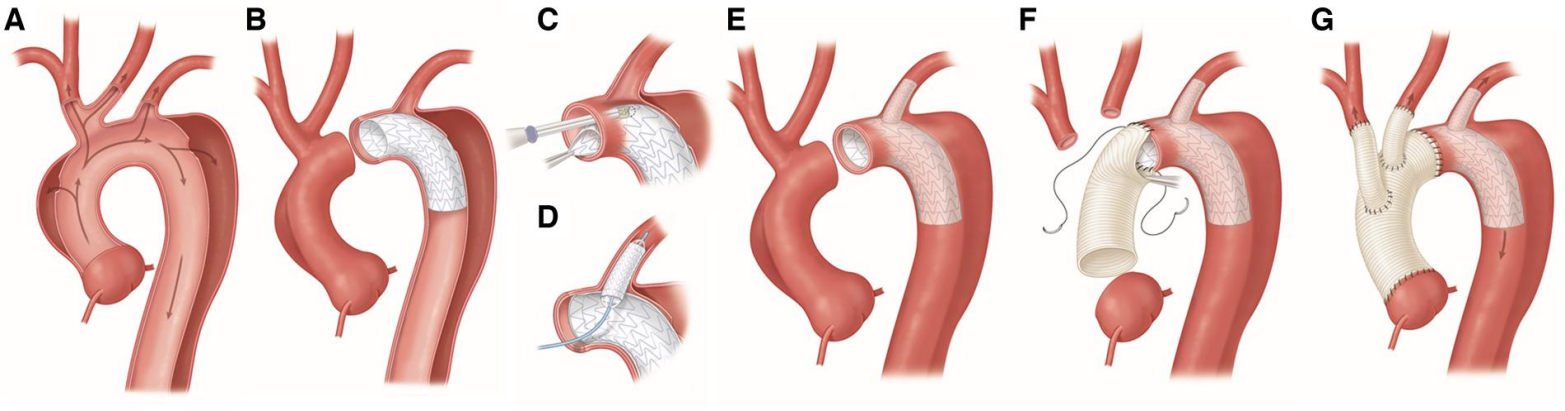
Preferred Technique for Branched Stented Anastomosis Frozen Elephant Trunk Technique. (A) Type A aortic dissection with entry tears in the ascending aorta and distal to the left subclavian artery. (B) Deployment of antegrade aortic stent graft in zone 2. (C) Direct fenestration of the stent graft at the location of the left subclavian artery. (D) Stenting the fenestration into the left subclavian artery. (E) Resection of the ascending aorta and base of the innominate and left common carotid arteries. (F) Distal anastomosis of the aortic graft to the proximal end of the stent graft at zone 2. (G) Anastomosis of the innominate and left common carotid branches to the ascending aortic graft

In both groups central cannulation was used when feasible, and cerebral debranching of the innominate and left common carotid was performed prior to circulatory arrest to allow for bilateral antegrade cerebral protection (ACP). If axillary cannulation was performed, unilateral ACP was done by clamping the innominate artery or bilateral ACP with a balloon catheter inserted directly into the left common carotid orifice at the discretion of the surgeon. Cerebral debranching was then performed after circulatory arrest and the distal anastomosis. In the HA-FET group, snares were placed circumferentially in zone 1 and zone 2 prior to circulatory arrest. The hybrid aortic arch device (Thoraflex) was deployed in zone 0, the snares cinched on the stent graft permitted lower body perfusion while the distal anastomosis was performed (Figure 1). After the proximal anastomosis was completed, the left subclavian artery was directly bypassed in the chest. In B-SAFER group an antegrade thoracic endovascular aortic repair (TEVAR) was deployed in zone 2, a fenestration was made for the left subclavian with a stent placed in it, then the distal anastomosis was performed (Figure 2).

Patients who underwent B-SAFER were cooled to moderate hypothermia for circulatory arrest with ACP. Patients who underwent HA-FET were maintained with mild hypothermia for circulatory arrest with ACP. The HA-FET technique was instituted in our centre in March 2023, prior to which our preferred technique for total arch replacement was the B-SAFER method. After the institution of the HA-FET technique the choice between the 2 techniques was at the discretion of the surgeon.

We excluded patients for HA-FET if there was (1) a hostile chest that required cooling for re-entry, (2) contained aortic rupture at the time of presentation, and (3) patients with a history of acute type A repair with a short ascending graft placed at the time of first operation,

### Study data

Demographic and preoperative comorbidities were collected on all patients via manual review of medical charts. The demographics of interest were age, sex, and body mass index. The comorbidities of interest were chronic lung disease, diabetes, renal failure, hypertension, tobacco use, alcohol use, prior myocardial infarction, prior cardiac surgery, and prior cerebrovascular accident. Indication for aortic arch replacement was categorized as rupture, aneurysm, or dissection. In addition, baseline left ventricular ejection fraction was collected.

Intraoperative data of interest were unilateral versus bilateral ACP, operative duration, nadir temperature, circulatory arrest duration, major concomitant procedures (ie, coronary artery bypass graft, aortic root replacement, aortic valve replacement, mitral valve repair or replacement, or valve-sparing root replacement).

### Outcomes

The primary study outcome was in-hospital mortality. Secondary outcomes examined were intraoperative blood product transfusion requirements, peak postoperative lactate, postoperative stroke, paralysis, renal failure, vocal cord paralysis, ventilator duration, and need for tracheostomy.

### Statistical analysis

Student *t*-tests were used to compare means for normally distributed continuous variables and Mann-Whitney U was used to compare medians for nonnormally distributed continuous variables. Fisher’s exact test was used to analyse categorical variables, as indicated. Statistical significance was considered at a *P*-value less than .05 and all tests were 2-tailed. Analyses were exploratory in nature.

## RESULTS

During the study period 61 patients underwent aortic arch replacement, 36 in the B-SAFER group and 25 in the HA-FET group. Both groups were of similar age and had similar comorbidities (Table 1). In the B-SAFER group 58.3% underwent total arch replacement in the setting of a type A aortic dissection compared to 60% in in the HA-FET group, with the majority of remaining patients undergoing a total arch replacement for an aneurysm (Table 2).

**Table 1.** Patient Demographics and Characteristics

	B-SAFER (*n* = 36)	HA-FET (*n* = 25)	*P*-value
Age	55 (49, 66)	63 (46, 67)	.56^a^
Sex (M)	25 (66.7)	21 (84)	.15^b^
BMI	28.7 (23.1, 35.15)	27.2 (23.4, 32.0)	.27^c^
Neurologic deficit on presentation	1 (2.8)	0 (0)	1.0^b^
Diabetes	4 (11.1)	7 (28)	.17^b^
Renal failure	2 (5.6)	3 (12)	.39^b^
Hypertension	31 (86.1)	17 (68)	.12^b^
Tobacco/drug use	13 (36.1)	3 (12)	.043^b^
Chronic lung disease	5 (13.9)	2 (8)	.69^b^
Prior CVA	4 (11.1)	0 (0)	.14^b^
MI	2 (5.6)	1 (4)	1.0^b^
Prior cardiac surgery	15 (38.9)	9 (36)	1.0^b^
Pre-LVEF	55 ± 7	54 ± 7	.71^c^
Pre-AI	1 (1, 2)	2 (1, 4)	.17^a^

Abbreviations: AI, aortic insufficiency; B-SAFER, branched stented anastomosis frozen elephant trunk; BMI, body mass index; CVA, cerebrovascular accident; LVEF, left ventricular ejection fraction; MI, myocardial infarction; M, male; HA-FET, hybrid arch frozen elephant trunk.

a Mann-Whitney U test.

b Fisher exact test.

c Student *t*-test.

**Table 2.** Indication for Surgery

	B-Safer (*n* = 36)	HA-FET (*n* = 25)
Ruptured PAU, *n* (%)	2 (5.6)	0 (0)
Aneurysm, *n* (%)	13 (36.1)	10 (40)
Dissection, *n* (%)	21 (58.3)	15 (60)
Type A, *n* (%)	17 (47.2)	12 (48)
Malperfusion syndrome, *n* (%)	4 (11.1)	3 (12)
Chronic arch, *n* (%)	4 (11.1)	3 (12)

Abbreviations: B-SAFER, branched stented anastomosis frozen elephant trunk; HA-FET, hybrid arch frozen elephant trunk; PAU, penetrating aortic ulcer.

The median operative duration (470 vs 462 minutes, *P* = .58), cardiopulmonary bypass (CPB) duration (229 vs 220 minutes, *P* = .67), and cross-clamp duration (116 vs 103 minutes, *P* = .94) were similar between the B-SAFER and HA-FET groups (Table 3). The B-SAFER group had a significantly lower median temperature for circulatory arrest compared to the HA-FET group (24 vs 32 °C, *P* < .001), as well as a longer median systemic circulatory arrest duration (40 vs 9 minutes, *P* < .001). In the B-SAFER group, 28% of patients underwent major concomitant procedures compared to 52% in the HA-FET group (*P* = .066). Both groups had similar 24-hour peak lactate values, and intraoperative transfusion requirements of red blood cells, plasma, and platelets. However, the B-SAFER group had a higher intraoperative requirement of cryoprecipitate than the HA-FET group (median 4 vs 2 units, *P* = .009).

**Table 3.** Intraoperative Details

	B-Safer (*n* = 36)	HA-FET (*n* = 25)	*P* value
Operative time	470 (377, 529)	462 (389, 506)	.58^a^
Operative time (no major concomitant)	464 (378, 512)	392 (350, 442)	.17^a^
CPB time	229 (207, 250)	220 (192, 271)	.67^a^
Cross-clamp time	116 (88, 161)	103 (83, 180)	.94^a^
Circ arrest time	40 (33, 54)	9 (6, 14)	<.001^a^
Circ arrest temp	23.8 (22, 25)	32 (25, 32.5)	<.001^b^
Bilateral ACP	28 (75)	18 (72)	1.0^c^
Major concomitant procedure	10 (27.8)	13 (52)	.066^c^
CABG	2 (5.6)	5 (20)	.11^c^
AVR	0 (0)	1 (4)	.41^c^
Root replacement	8 (21.6)	8 (32)	.55^c^
VSRR	1 (2.8)	2 (8)	.56^c^
Neo-media root reconstruction	8 (22.2)	2 (8)	.18^c^
No root pathology	19 (52.8)	12 (48)	.80^c^
Peak lactate (24 hours)	6.2 (4.9, 11.1)	6.5 (4.5, 10.3)	.26^a^
RBC (units)	3 (1, 6)	4 (1, 6)	.65^a^
Platelets (units)	2 (2, 5)	3 (1, 4)	.74^a^
Plasma (units)	6 (2, 8)	5 (1, 8)	.48^a^
Cryoprecipitate (units)	4 (2, 4)	2 (0, 2)	.009^a^

Abbreviations: ACP, antegrade cerebral perfusion; AVR, aortic valve replacement; B-SAFER, branched stented anastomosis frozen elephant trunk; CABG, coronary artery bypass graft; CPB, cardiopulmonary bypass; HA-FET, hybrid arch frozen elephant trunk; RBC, red blood cells; VSRR, valve-sparing root replacement.

a Mann-Whitney U test.

b Student *t*-test.

c Fisher exact test.

Both groups had similar postoperative length of stay (Table 4). The in-hospital mortality rate for B-SAFER was 8% (*n* = 3) compared to 4% (*n* = 1) for HA-FET (*P* = .64). In the B-SAFER group 2 patients (5.6%) had a postoperative neurologic deficit compared with 1 patient (4%) in the HA-FET group (*P* = 1). No patients in either group had postoperative paralysis. Both groups had similar rates of acute kidney injury, vocal cord paralysis, and requirement for tracheostomy. Three patients (8.3%) in the B-SAFER group had a new postoperative requirement for dialysis compared to 3 patients (12%) in the HA-FET group (*P* = .68).

**Table 4.** Postoperative Outcomes

	B-Safer (*n* = 36)	HA-FET (*n* = 25)	*P* value
Hospital LOS	14 (10, 21)	20 (14, 27)	.057^a^
ICU LOS	6 (3, 9)	9 (6, 14)	.067^a^
In-hospital mortality	3 (8.3)	1 (4)	.64^b^
Postneurologic deficit	2 (5.6)	1 (4)	1.0^b^
Postparaplegia	0 (0)	0 (0)	1.0^b^
Postdialysis	3 (8.3)	3 (12)	.68^b^
Post-Op vocal cord paralysis, *n* (%)	3 (8.3)	2 (8)	1.0^b^
Post-Op vent duration (hours), mean ± SD	45.5 ± 43.1	50.4 ± 44.9	.020^c^
Re-intubation, *n* (%)	1 (2.8)	3 (12)	.30^b^
Tracheostomy, *n* (%)	2 (5.6)	2 (8)	1.0^b^

Abbreviations: AKI, acute kidney injury; B-SAFER, branched stented anastomosis frozen elephant trunk; ICU, intensive care unit; LOS, length of stay; HA-FET, hybrid arch frozen elephant trunk.

a Mann-Whitney U test.

b Fisher exact test.

c Student *t*-test.

Both groups had similar rates of aortic reinterventions involving the arch or proximal descending (25% vs 24%, *P* = 1) for B-SAFER and HA-FET groups, respectively. Among the B-SAFER cohort, 2 required endovascular treatment of a left subclavian 1 C endoleak, 5 required TEVAR extensions in zone 3, 1 required a zone 1 TEVAR for a 1 A endoleak, and 1 required an open thoracoabominal aortic replacement. In the Thoraflex group, 6 required TEVAR extensions starting in zone 3 with 1 also requiring coiling of left subclavian artery from a type II endoleak.

## DISCUSSION

Our cohort demonstrated that the B-SAFER and HA-FET techniques for total arch replacement have comparable short-term outcomes, including mortality, stroke, and paraplegia rates. The in-hospital mortality and incidence of permanent neurologic dysfunction were relatively low and comparable between the 2 groups. Of the 3 mortalities in the B-SAFER group, 1 had signs of cerebral malperfusion prior to the operation in the setting of a type A and postoperatively had a devastating neurologic injury, 1 had overwhelming sepsis and multisystem organ failure after a type A repair with a preoperative arrest, and the third had overwhelming sepsis and multisystem organ failure after a reoperative aortic root and arch replacement. The death in the HA-FET group was due to sepsis and multisystem organ failure in the setting of a redo arch replacement for a chronic arch dissection. These results suggest that both approaches are viable options for aortic arch repair, each with unique advantages that can be tailored to individual patient anatomy and surgical needs.

Roselli et al.s’ early results of the B-SAFER technique were published as a prospective, single-centre study enrolled 178 patients undergoing B-SAFER for various indications, including acute aortic syndrome, chronic aortic dissection, degenerative aortic aneurysm, and congenital aortic arch disease. The study reported an operative mortality rate of 5.6%, with disabling stroke occurring in 2.9% of patients and paraparesis in 0.6% of patients. We noted similar outcomes in our retrospective B-SAFER cohort indicating a consistent and sound surgical approach by our team. These outcomes highlight the safety and reproducibility of the B-SAFER technique in a complex and heterogeneous patient cohort, supporting its generalizability and use in managing intricate aortic arch pathologies.^3^

In a multicentre trial led by Coselli et al., the Thoraflex device has demonstrated promising results in the United States for total aortic arch replacement. In this prospective trial involving 12 US sites, 65 patients underwent Thoraflex Hybrid repair for various aortic pathologies. At 1 year, the study reported a freedom from major adverse event rate of 81% in the primary group, with a 30-day mortality rate of 11%, a stroke rate of 5%, and permanent paraplegia/paraparesis in 5% of patients. We also noted similar, if not slightly better outcomes, in our HA-FET cohort, though differences in a standardized surgical approach at our centre versus varying approaches in a multicentre study may account for this. These findings support the use of the Thoraflex Hybrid device for extensive thoracic aortic repair, highlighting its potential to facilitate single-stage repair while providing a stable landing zone for subsequent endovascular procedures. However, long-term data are needed to further assess the durability of repairs with this device.^6^

In a meta-analysis Tian et al. found a strong correlation between mortality and the durations of CPB, cross-clamp, and circulatory arrest.^7^ In our study, both the HA-FET group and the B-SAFER group had a similar CPB and cross-clamp durations, despite significantly shorter circulatory arrest durations in the HA-FET group. This is likely due to the higher incidence of major concomitant procedures in the HA-FET group. Our group found this technique enabled for efficient management of complex cases without prolonging operative times. Both groups also had similar mortality in our study despite the nearly 4-fold decrease in circulatory arrest duration in the HA-FET group. However, this may be due to our limited cohort size and with a larger sample size differences in mortality may emerge.

The French national registry study, conducted between 2016 and 2019, evaluated the Thoraflex Hybrid prosthesis in emergency FET procedures for 109 patients, finding an in-hospital mortality rate of 20%-22% and an 8% paraplegia rate. Outcomes were consistent across various centres, highlighting the importance of spinal cord ischaemia prevention.^4^ Additionally, a separate study from 2016 to 2021 using a simplified delivery technique (SD-FET) allowed for distal suturing under moderate hypothermia or normothermia, significantly reducing circulatory arrest time (4.4 ± 2.4 minutes). This study showed lower in-hospital mortality for SD-FET (11.9%) compared to the conventional approach (19.2%) and similar rates of postoperative stroke and spinal cord injury. This suggests that mild or moderate hypothermic approaches using Thoraflex can be effective and safe for aortic arch pathologies.^5^ We had no occurrences of paraplegia in our HA-FET cohort. This may be due to proximalizing the deployment of the FET to zone 0, thus resulting in less coverage of the descending thoracic aorta.

While other strategies have been reported that allow for mild hypothermia or normothermia and minimize or even eliminate circulatory arrest duration, they can add significant complexity to the operation. Eusanio et al. reported a technique utilizing axillary and femoral arterial cannulation with placement of a retrograde TEVAR and endoballoon prior to performing the distal anastomosis to eliminate circulatory arrest time.^8^ However, this approach requires fluoroscopy, comfort with an endoballoon, and retrograde flow via the femoral artery, which has been associated with worse outcomes, particularly in the setting of type A dissections.^9^

Our group was encouraged by the results from the SD-FET and the relative simplicity, as such we sought to perform the HA-FET technique with the thought that we may further improve outcomes in our total arch operation. However, despite a significantly shorter circulatory arrest time at mild hypothermia, the HA-FET group had comparable mortality to that observed with a longer duration of circulatory arrest at moderate hypothermia in the B-SAFER group. While this may be due to differences in the complexity of concomitant procedures performed, this observation indicates that both approaches, utilizing ACP, maintained similar levels of patient safety and efficacy. Similarly, the comparable results in terms of neurologic outcomes, despite the differences in circulatory arrest duration and temperature management, highlight the effectiveness of both techniques in managing aortic arch pathologies. We have not found the dissection and release of aortic arch, even in redo settings to be prohibitive to the HA-FET approach with applying the snares. Though we acknowledge the small sample size, there may be scenarios that pose more challenging. If this scenario is encountered or anticipated, it may be reasonable to further cool the patient and perform a running distal anastomosis under lower body circulatory arrest.

Hypothermia has served as a protective measure during circulatory arrest for the both the brain and visceral organs. However, recent practice has shifted away from deep hypothermia towards progressively higher temperatures, particularly with the use of ACP.^^10–12^^ With evolving technology and surgical technique, shorter durations of circulatory arrest are required and permit less cooling than was historically required. In addition, there has been some suggestion that the degree of hypothermia is correlated to perioperative coagulopathy.^13^^,^^14^ We found no significant difference in transfusion rates between the B-SAFER and the HA-FET groups in this cohort. This finding could be confounded by the higher incidence of major concomitant procedures in the HA-FET group. However, the lower use of cryoprecipitate in the HA-FET group suggests a possible trend towards reduced bleeding risk, which merits further investigation.

Our study is limited by its nature as a single-centre nonrandomized retrospective study and small sample size. As the 95% confidence intervals were not adjusted for multiple comparisons, inferences drawn from them may not be reproducible. Cases that necessitated cooling for sternal re-entry or contained rupture did not permit the HA-FET technique and as such were excluded, which may introduce bias. However, these were a small minority of cases. In addition, the operations performed were not contemporaneous, with the HA-FET technique being utilized after the implementation of the B-SAFER technique at our centre. However, after implementation of the HA-FET technique, the B-SAFER technique was still used by our group and is still a suitable option, particularly in the absence of availability of appropriate hybrid arch devices. In addition, the majority of cases was performed by 2 surgeons specializing in aortic pathology. As the choice between B-SAFER and HA-FET was at surgeon discretion, the groups may differ in baseline characteristics and unmeasured confounders could affect the observed outcomes.

In conclusion, both the B-SAFER and HA-FET techniques offer safe and effective options for total arch replacement. Further studies with larger cohorts are necessary to fully understand the long-term outcomes and to determine the optimal surgical approach for different patient populations. The continued evolution of aortic arch repair techniques and hybrid aortic arch devices has the potential to significantly enhance patient care and outcomes in the future.

## Data Availability

The data underlying this article will be shared on reasonable request to the corresponding author.
